# Fatigue Growth Behaviour of Two Interacting Cracks with Different Crack Offset

**DOI:** 10.3390/ma12213526

**Published:** 2019-10-28

**Authors:** Huijin Jin, Bing Cui, Ling Mao

**Affiliations:** 1 State Key Laboratory of Nonlinear Mechanics, Institute of Mechanics, Chinese Academy of Sciences, Beijing 100190, China; 2 School of Materials Science and Engineering, Anhui University of Technology, Ma’anshan 243000, China; 3 School of Materials Science and Engineering, Beihang University, Beijing 100191, China

**Keywords:** non-collinear cracks, finite element analysis, fatigue crack growth, interacting behaviour

## Abstract

Under cyclic fatigue load, multiple cracks would significantly deteriorate the service life of the components with respect to the case of a single crack owing to the crack interaction. The present study aims to explore the effect of crack interaction on the fatigue growth behaviour of samples with different crack offset. In this study, fatigue crack growth tests were performed for samples containing a single crack and non-collinear cracks of different crack offset in an aluminum–lithium alloy. It was shown that the two facing non-collinear cracks changed their growth direction when the cracks were overlapped, resulting in load mode transfers from mode I to I + II mixed mode. Then, the interaction behaviour was studied by establishing the finite element models to calculate the stress intensity factor K of samples with different crack offset. The results indicated that the K decreased, largely owing to the shielding effect as the two cracks overlapped, leading to retardation of crack growth in the position of overlap, especially for the specimens with a small crack offset. It was also shown that the interaction effect could change from positive to negative during the process of the multiple cracks’ growth, thus leading to the acceleration or deceleration of crack growth rates, suggesting that the influence of interaction on cracks’ growth behaviour could vary with the different stages of crack growth.

## 1. Introduction

Multiple cracks may be initiated and propagated owing to fatigue and corrosion in structural components, especially at multiple riveted locations [1,2,3]. During the growth process of a crack, the interaction of multiple cracks might occur when a specific condition is reached, which could have a large impact on crack growth behaviour [4,5]. The interaction might cause the increase of the crack growth rate when the adjacent cracks merge, resulting in the crack growth life being greatly reduced. Although many investigations on the influence of interaction on the cracks’ growth behaviour have been conducted [4,6,7,8,9,10,11], detailed research on the effect of the interaction on multiple cracks’ growth behaviour of specimens with different crack offset is very limited. 

The multiple crack interactions could affect the stress fields around the crack tip, which could change the stress intensity factor *K* on the crack tip [12,13], thus leading to the acceleration or retardation of crack growth rates. The stress intensity factor *K* is usually calculated by the finite element method and theoretical analysis method. Han et al. [14] adopted the finite element approach to calculate the *K* of parallel cracks, indicating that the relative positions between cracks have a great effect on the interactions. Zhao et al. [15] propose a modified analytical solution to obtain the *K* of multiple hole-edge cracks. The authors introduced the approximate superposition method to add some other impacts to the equivalent crack in the complex variable function. The method showed that the modified method has good agreement with the finite element method. Jin et al. [16] proposed an improved *K* method to study the interacting behaviour of double collinear cracks growth. The improved *K* method was calculated using the net section stress σ_n_ instead of the applied remote stress σ in the equation of stress intensity factor, showing that the method could accurately describe the collinear multiple cracks growth behavior.

During the multiple cracks’ growth processes, the interaction could change the crack propagation directions, thus affecting the crack propagation rates. The experimental and numerical analysis studies [17,18] showed that the multiple cracks’ propagation rate could be accelerated by comparing with a single crack as the neighboring cracks are merged, while the cracks may also be retardated or stationary owing to the impact of stress shielding. Also, the multi-cracks may grow under the I + II mixed mode loading condition instead of the mode I condition, because of the change of crack growth directions caused by interactions. In linear elastic fracture mechanics (LEFM), mode I and mode II are the two basic modes of crack deformation: mode I (opening), the growth directions of the crack are perpendicular to the normal stress; and mode II (sliding), the growth directions of the crack are parallel to the shearing stress. In addition, I + II mixed mode includes mode I (opening) and mode II (sliding). With the increase in the crack interactions, the extent of the cracks’ deflection is increased owing to the increase of mode II [19]. However, some characteristics about crack growth deceleration have been insufficiently investigated, especially for the effect of the crack offset on multi-cracks’ growth rates.

In this study, numerical computations were carried out to evaluate the stress intensity factor of interacting cracks with different crack offset. Then, the effect of cracks’ interaction on the crack propagation bahaviour was studied by the fatigue crack growth tests for samples containing non-collinear cracks of different crack offset. The focus was on how the crack growth rates of samples with different crack offset were affected by crack interactions during the process of the multiple cracks’ growth.

## 2. Experimental Methods

**Figure 1:**
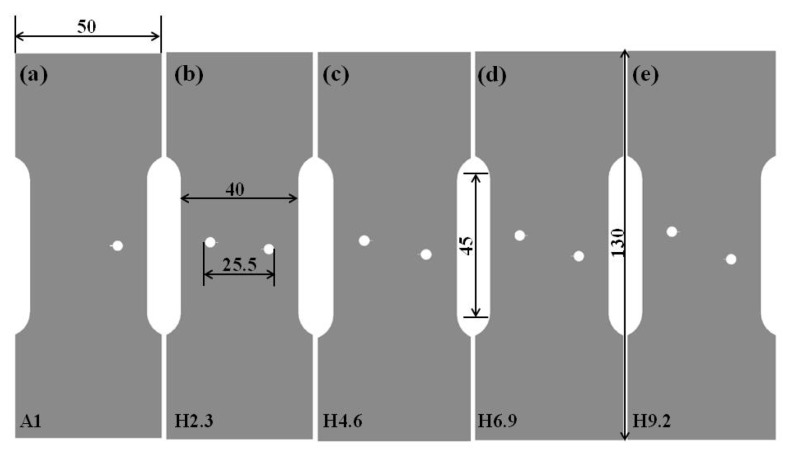
Sample geometry (all dimensions in mm): (**a**) specimen A1 containing a single crack; (**b**) specimen H2.3 (initial condition: crack offset H = 2.3 mm), (**c**) specimen H4.6 (initial condition: H = 4.6 mm), (**d**) specimen H6.9 (initial condition: H = 6.9 mm), (**e**) specimen H9.2 (initial condition: H = 9.2 mm). The thickness of all specimens is 2 mm.

The crack propagation tests of 2060 aluminum–lithium alloy specimens with a single crack and non-collinear cracks of different crack offset were performed under the fatigue load at room temperature (297 K). The alloy had a chemical composition of 3.95 Cu, 0.75 Li, 0.3 Mn, 0.85 Mg, 0.4 Zn, 0.11 Zr, 0.25 Ag, and balance Al in wt.%. The crack configurations of different samples are shown in Figure 1. All samples were cut along the longitudinal (L)–transverse (T) direction. The mechanical slits were cut at the edge of the holes using wire-electrode cutting and the diameter of the molybdenum wire was 0.1 mm. For all samples, the initial crack length of each crack was the sum of the slit length (1 mm), the hole diameter (3.5 mm), and the fatigue precracking length (0.5 mm). 

A digitally-controlled material test system (MTS, 810, MTS Systems Corporation, Minnesota, USA) 100 kN force cyclic test machine was used to perform the fatigue tests. The testing-frequency was 8 Hz and the cyclic stress ratio was R = 0.1. The maximum and minimum fatigue loads of all samples were the same, 7 kN and 0.7 kN, respectively. To ensure the reliability of the tests, at least three identical specimens were measured for each type of sample geometry. A travelling microscope was adopted to measure the crack length in a certain cycle number. When the specimen was fractured, the fatigue tests were finished. 

**Figure 2:**
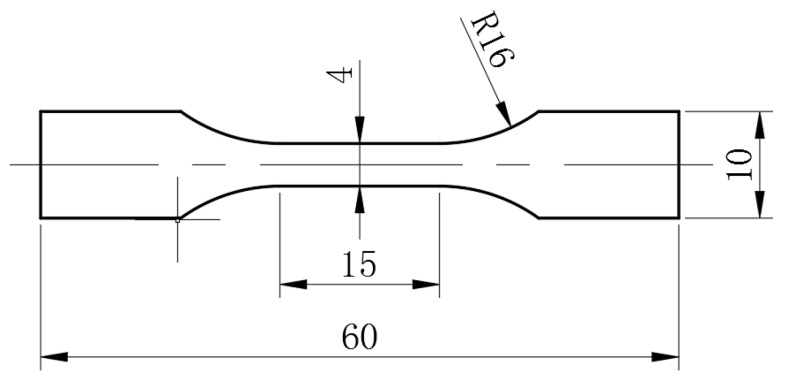
Shape and dimensions of tensile specimens (all dimensions in mm).

The tensile properties of samples were measured on the Instron 8801 type −100 kN testing machine Instron Corporation, Boston, UA) at a strain rate of 0.1 mm/min, and the shape and dimensions of tensile specimens are shown in Figure 2. Five tensile specimens were tested to obtain the average values of the Young’s modulus E, yield strength Re, elongation ε, and ultimate strength Rm; these values were 74.09 GPa, 480.44 MPa, 15.19%, and 529.7 MPa, respectively.

## 3. Finite Element Models

**Figure 3:**
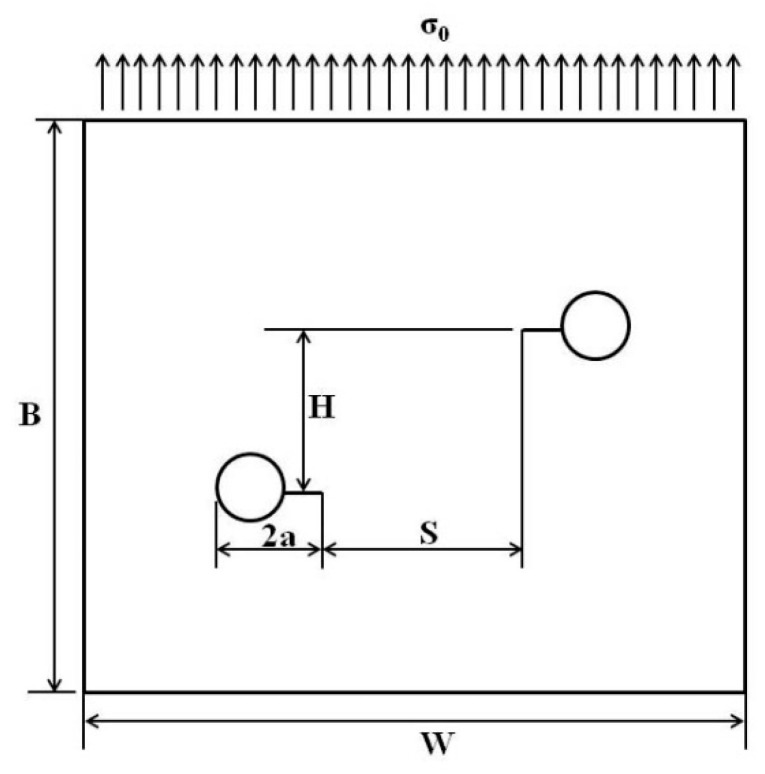
Geometry of a plate with two non-collinear through cracks.

**Figure 4:**
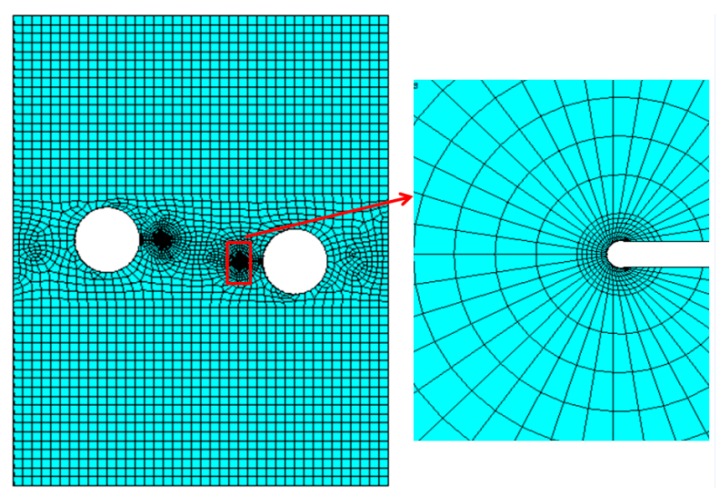
An example finite element mesh.

The finite element models were created to numerically calculate the K for two interacting non-collinear cracks. The horizontal distance S and crack offset H were defined in Figure 3. The plate with two through-thickness parallel cracks was subjected to the tensile loading, σ_0_. The software ABAQUS (version 6.5, Dassault Simulia Corporation, Rhode Island, USA)) [20] was adopted to carry out the finite element analysis. The *K* was derived from the energy release rate, which was obtained by the virtual crack extension integral method [21]. The method considers a path Γ that encloses the crack tip and with initial and final points, which lie on the two crack faces; this integral can characterize the energy release associated with the crack growth, as follows:

(1) $$J=\int_{\Gamma }\left(Udy-t\frac{\partial d}{\partial x}ds\right),$$

where *U* is the strain energy density, *t* the traction vector on a plane defined by the outward normal, *d* the displacement vector, and *ds* the element of arc along the path *Γ*. This integral method is used to evaluate contour integrals automatically in the finite element code ABAQUS. Therefore, the stress intensity factor *K* is directly related to the *J*-integral, which is expressed as follows:

(2) $$K=(\frac{8GJ}{1+\kappa }{)}^{1/2},$$

where *G* is the shear modulus, κ is a function of Poisson’s ratio, κ=(3−υ)/(1+υ) in plane stress state, κ=3−4υ in plane strain state and υ is the Poisson’s ratio. To confirm of the accuracy of *K*, a relatively fine mesh was adopted on the crack tip. An example of the finite element mesh for a type of crack geometry is presented in Figure 4. The mesh contains approximately 16,292 eight-noded solid elements for the crack geometry of S = 15.5 mm, H = 2.3 mm, and 2a = 5 mm.

## 4. Results and Discussion

### 4.1. Simulation Results of Stress Intensity Factor

**Figure 5:**
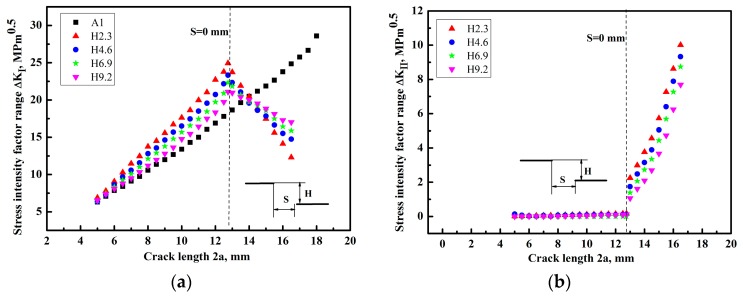
The stress intensity factor range (**a**) Δ*K_I_* and (**b**) Δ*K_II_* changing with the crack growth length 2a.

**Figure 6:**
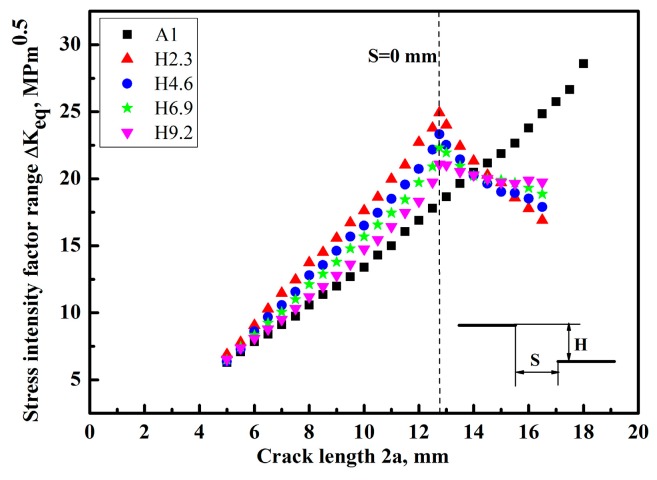
The Δ*K_eq_* varying with the crack growth length 2a.

The finite element method was used to calculate the *K* at the tip of the crack for different crack lengths. This method can derive the accurate *K* by considering the cracks’ interaction [22,23]. Figure 5a,b and Figure 6 show the results of the mode I stress intensity factor range Δ*K_I_*, the mode II stress intensity factor range Δ*K_II_*, and the equivalent stress intensity factor range Δ*K_eq_*, along with the crack propagation length 2a, respectively. For the Δ*K_eq_*, several equations were presented under the I + II mixed mode load [24,25]. In this study, the equation put forward by Tanaka et al. [26] was used, which is defined as follows:

(3) $$\Delta K_{\mathrm{eq}}={\left[\Delta K_{I}^{4}+8\Delta K_{\mathrm{II}}^{4}\right]}^{0.25},$$

where Δ*K_eq_* is the equivalent stress intensity factor range, Δ*K_I_* is the mode I stress intensity factor range, and Δ*K_II_* is the mode II stress intensity factor range. 

It can be seen from Figure 5a that the Δ*K_I_* was increased with the increase of crack growth length 2a for the specimen A1 having a crack. Compared with the sample A1, the Δ*K_I_* of specimens H2.3, H4.6, H6.9, and H9.2 with different crack offset distance was firstly increased until the inner crack tips of two cracks were overlapped, and then Δ*K_I_* was decreased with the increase of the crack length owing to the shielding effect. It should be noted in Figure 5b that the values of Δ*K_II_* were almost zero at first, when the two facing cracks were overlapped, and Δ*K_II_* was added gradually with the increase of the crack growth length. Although values of Δ*K_II_* were small compared with Δ*K_I_*, it could affect the crack growth directions. Moreover, Δ*K_II_* was gradually increased as the magnitude of overlapping was added owing to the interaction effect. The change trend of Δ*K_eq_* versus 2a (Figure 6) was similar to that of Δ*K_I_* versus 2a. The Δ*K_I_* and Δ*K_eq_* were increased firstly, and then decreased along with the crack length 2a in the region of overlap, finally reaching a very low value, which meant that the multiple crack growth rate for two non-collinear cracks with different crack offset was mainly dominated by Δ*K_I_*, even under the I + II mixed mode load. 

On the other hand, as shown in the case of H9.2, it had a relatively low stress intensity factor due to a large crack offset comparing with the specimens H2.3, H4.6, and H6.9, which was mainly attributed to the small interactions of cracks. For the sample H2.3 with relatively small crack offset, the values of Δ*K_I_* and Δ*K_II_* were larger compared with other samples H4.6, H6.9, and H9.2 before the cracks crossed, which is because of the big interaction. For the specimens H2.3, H4.6, H6.9, and H9.2 with non-collinear cracks, the stress intensity factor at the overlapped position was greatly reduced owing to the negative interaction influence when the two cracks were overlapped, which could lead to the retardation of crack growth at the overlapped position. Moreover, the negative interaction is more pronounced with the increase of overlap degree due to the stress-shielding effect. Therefore, it can be concluded that the interaction effect changed from the positive interaction to the negative interaction during the process of cracks’ growth. 

### 4.2. Multi-Cracks Fatigue Growth Path

**Figure 7:**
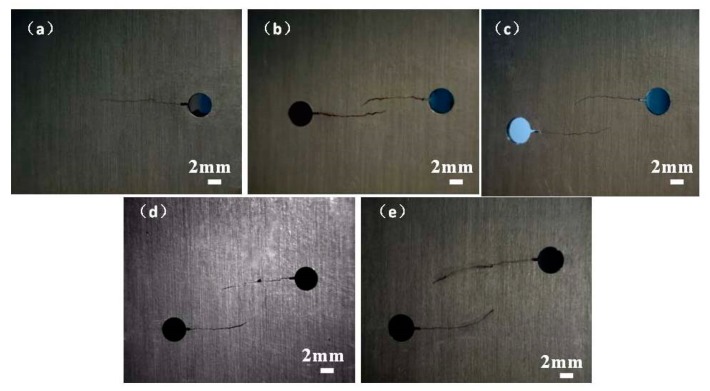
Crack propagation paths. (**a**) A1 with a single crack, (**b**) specimen H2.3 (initial condition: crack offset H = 2.3 mm), (**c**) specimen H4.6 (initial condition: H = 4.6 mm), (**d**) specimen H6.9 (initial condition: H = 6.9 mm), (**e**) specimen H9.2 (initial condition: H = 9.2 mm).

Crack growth paths for samples A1 having a single crack and samples H2.3, H4.6, H6.9, and H9.2 with different crack offsets of H = 2.3 mm, 4.6 mm, 6.9 mm, and 9.2 mm, respectively, are shown in Figure 7. For sample A1, it can be found from Figure 7a that the new crack was initiated at the notch and grew perpendicular to the fatigue load. For the double non-collinear cracks, at the early stage of the crack propagation, the two cracks grew perpendicularly to the fatigue loading direction, similar to the specimen A1 with a single crack. However, when the cracks overlapped, the growth direction of two facing cracks began to change so that they were closed to each other, causing that the crack modes transfer from mode I to I + II mixed mode, as shown in Figure 7b–e. Moreover, the curvature of cracks occurred not only affecting *K_I_*, but also causing the increase of *K_II_* around the crack fronts as the cracks grew. It was indicated when the double cracks approached one another, the stress states at the tip of crack would change as a result of the effect of the interaction. Therefore, during the process of the cracks’ growth, the stress states changed from mode I to I + II mixed mode. 

**Figure 8:**
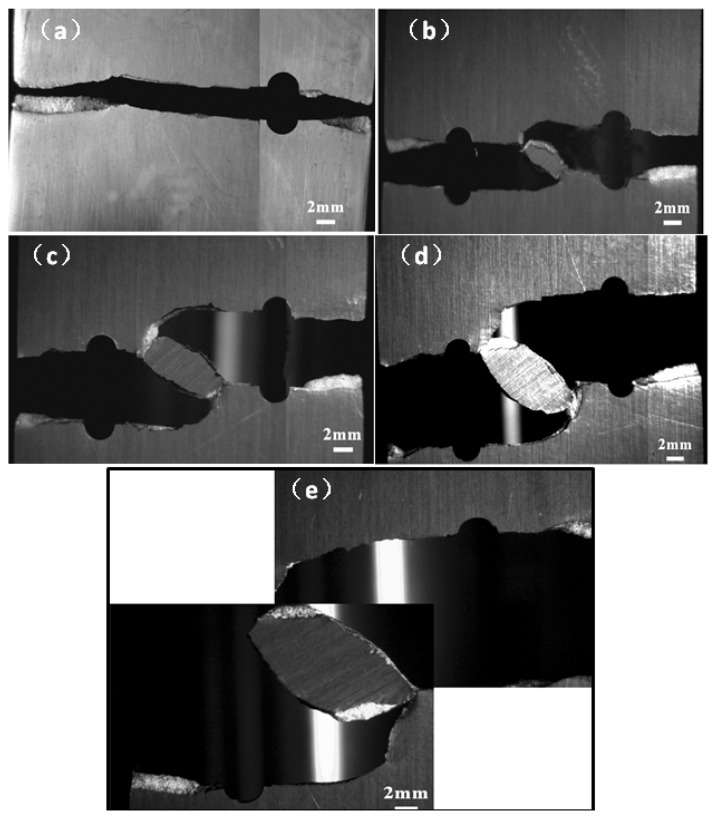
The fractured specimens for (**a**) A1 with a single crack, (**b**) specimen H2.3 (initial condition: crack offset H = 2.3 mm), (**c**) specimen H4.6 (initial condition: H = 4.6 mm), (**d**) specimen H6.9 (initial condition: H = 6.9 mm), (**e**) specimen H9.2 (initial condition: H = 9.2 mm).

The fractured sample with a crack is displayed in Figure 8a and two non-collinear cracks with different crack offset distance H are presented in Figure 8b–e. It can be found that the fracture pattern for specimens H2.3, H4.6, H6.9, and H9.2 with different crack offset distance is nearly similar, that is, firstly the mode I fracture and then the I + II mixed mode fracture. However, it is seen that the sizes of the remaining ligament between two cracks are added with the increase of crack offset H. It is also seen that the crack growth length of two facing cracks was almost similar owing to the same initial notch lengths. When the crack deviation of adjacent crack tips begins to occur, the growth rates of two cracks will be decelerated because of the stress shielding effect. In all cases of the specimens with different H, the double facing cracks did not merge until the sample was fractured, as seen in Figure 8b–e. That is, the cracks coalesced along with the rupture of the specimens.

### 4.3. Multi-Cracks’ Fatigue Growth Rate

**Figure 9:**
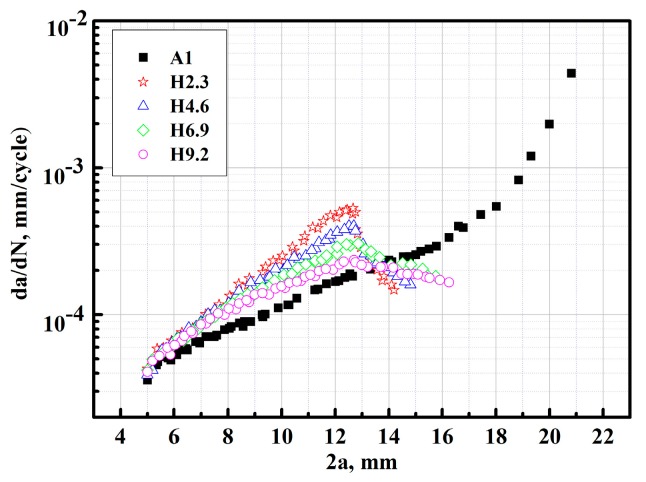
The crack propagation rates plotted with fatigue crack length for specimens H2.3, H4.6, H6.9, and H9.2 compared with specimen A1.

The relationship of crack growth rates da/dN and the fatigue crack lengths 2a for specimens H2.3, H4.6, H6.9, and H9.2 compared with specimen A1 containing a crack was shown in Figure 9. It can be seen that the da/dN were continuously increased with fatigue crack lengths for the specimen with a single crack. However, for specimens H2.3, H4.6, H6.9, and H9.2 with different crack offset distance H, the da/dN were firstly increased, and then decreased with fatigue crack lengths. From Figure 9, it can be seen that when the crack length was greater than about 6.5 mm, the crack growth rates of the samples H2.3, H4.6, H6.9, and H9.2 were faster than that of the specimen A1 containing a single crack in the middle and late stages during the crack steady growth process, which means that the presence of multiple cracks accelerated the da/dN of samples H2.3, H4.6, H6.9, and H9.2. Even for H9.6 with the greater H, the da/dN was faster than that of the specimen A1 containing a single crack. Also, when the H became small, the acceleration effect became large. However, when the inner crack tips of the double facing cracks were overlapped—and at this moment, the two facing cracks began to change their growth direction—it can be observed from Figure 9 that the da/dN of samples H2.3, H4.6, H6.9, and H9.2 were reduced greatly owing to the decreased driving force of crack growth caused by the cracks’ shielding effect. In this case, the retarding effect of crack propagation exceeded the acceleration effect, and the effect of interaction on crack propagation varied from positive to negative. Furthermore, the shielding effect became greater with the increase of overlapping extent, which caused the lower crack grow rate. This suggests that, owing to the change of interaction effect, the effect of cracks interacting on multiple crack growth behaviour should be evaluated by considering the whole process of crack propagation for non-collinear cracks with different crack offset H. Thus, the influence of interaction on multiple cracks’ growth behaviour depended greatly on the different stages of crack propagation. Therefore, this study could provide the theoretical basis and technical references for fatigue life prediction and the safety assessment of components containing non-collinear multi-cracks. 

## 5. Conclusions

The influence of the interaction on multiple cracks’ fatigue growth behaviour for the specimens with different crack offset compared with the specimen containing a single crack was studied by experimental and numerical investigations. The conclusions are as follows:

(1) The double non-collinear cracks could change their growth direction when the two cracks were close to each other, leading to the change of load mode from mode I to I + II mixed mode as a result of the interaction. Moreover, Δ*K_II_* affecting the growth directions of the crack tip was gradually increased as the magnitude of overlapping was added.
(2) Finite element analysis shows that the Δ*K_I_* and Δ*K_eq_* of specimens with different crack offset were reduced greatly when the two cracks were overlapped, and the specimens with a large crack offset distance have a relatively low Δ*K_I_* and Δ*K_eq_*, owing to a small interaction. Δ*K_I_* and Δ*K_eq_* are the main parameters of the crack growth driving force, and the experimental results of crack growth rate have a similar change trend to that of Δ*K_I_* and Δ*K_eq_* by numerical analysis.
(3) The cracks’ interaction has a positive or negative effect on the stress intensity factor of specimens with two non-collinear cracks, which depended on the relative position of the two cracks, thus leading to the acceleration or deceleration of crack growth rate compared with the specimen with a single crack.
